# Intranasal insulin modulates cerebrospinal fluid markers of neuroinflammation in mild cognitive impairment and Alzheimer’s disease: a randomized trial

**DOI:** 10.1038/s41598-022-05165-3

**Published:** 2022-01-25

**Authors:** Derek Kellar, Thomas Register, Samuel N. Lockhart, Paul Aisen, Rema Raman, Robert A. Rissman, James Brewer, Suzanne Craft

**Affiliations:** 1 Department of Internal Medicine–Geriatrics, Wake Forest School of Medicine, Winston-Salem, NC USA; 2 Alzheimer’s Therapeutic Research Institute, University of Southern California, San Diego, USA; 3 Department of Neurosciences, University of California, San Diego, La Jolla, USA

**Keywords:** Immunology, Neuroscience, Biomarkers, Diseases, Medical research, Neurology

## Abstract

Intranasal insulin (INI) has shown promise as a treatment for Alzheimer’s disease (AD) in pilot clinical trials. In a recent phase 2 trial, participants with mild cognitive impairment (MCI) or AD who were treated with INI with one of two delivery devices showed improved cerebral spinal fluid (CSF) biomarker profiles and slower symptom progression compared with placebo. In the cohort which showed benefit, we measured changes in CSF markers of inflammation, immune function and vascular integrity and assessed their relationship with changes in cognition, brain volume, and CSF amyloid and tau concentrations. The insulin-treated group had increased CSF interferon-γ (p = 0.032) and eotaxin (p = 0.049), and reduced interleukin-6 (p = 0.048) over the 12 month trial compared to placebo. Trends were observed for increased CSF macrophage-derived chemokine for the placebo group (p = 0.083), and increased interleukin-2 in the insulin-treated group (p = 0.093). Insulin-treated and placebo groups showed strikingly different patterns of associations between changes in CSF immune/inflammatory/vascular markers and changes in cognition, brain volume, and amyloid and tau concentrations. In summary, INI treatment altered the typical progression of markers of inflammation and immune function seen in AD, suggesting that INI may promote a compensatory immune response associated with therapeutic benefit.

## Introduction

Alzheimer’s disease (AD) is a neurodegenerative disorder that is the leading cause of dementia and that incurred an estimated cost of $305 billion in 2020 alone^1^. As aging is the most common risk factor for the disease, an increasingly aged population will exacerbate this burden, with cost estimates rising to over $1 trillion by 2050^1^. Alzheimer’s disease pathology is characterized by the aggregation of amyloid beta (Aβ) into extracellular plaques and hyperphosphorylated tau into intracellular neurofibrillary tangles (NFT). For decades, therapeutic approaches have focused on elimination or reduction of these two proteins in the brain; however, successful prevention of cognitive decline using these strategies has been limited and controversial^2^. There remains a clear and urgent need to develop a deeper understanding of the comprehensive brain environment throughout the course of AD and to find a way to alter this trajectory.

A promising area of research focuses on prevention of cognitive decline by restoring normal brain insulin function through the administration of intranasal insulin (INI)^3^. Several recent reviews have implicated insulin signaling and insulin resistance in AD^3–5^. Insulin may directly affect AD pathology as it impacts production and clearance of the Aβ peptide and prevents hyperphosphorylation of tau^4^. There is evidence suggesting impaired insulin function, a condition known as insulin resistance, can predict Aβ accumulation up to 15 years in advance^6^. Insulin resistance also correlates with worse cognition and higher concentrations of both hyperphosphorylated and total tau in healthy adults^7^. Insulin has many additional effects of relevance to AD and other neurological disorders. It modulates astrocyte response to inflammatory signaling, resulting in altered cytokine secretion that can elicit an anti-inflammatory response^8,9^. Insulin inhibits apoptosis and, through its AKT pathway, may promote oligodendrocyte proliferation, survival and myelination^10,11^.

A recent large 18-month phase 2 clinical trial examined the effects of INI on cognition, function, brain structure and CSF AD biomarkers in participants with MCI or AD^12^. Two cohorts received insulin (40 IU regular insulin) or placebo daily using different intranasal delivery devices. Treatment with the device used by the primary cohort yielded no significant difference in cognition or CSF biomarkers between the insulin and placebo groups. In prespecified analyses of the cohort that used the second device, however, insulin treatment was associated with better performance compared with placebo on the primary outcome, the Alzheimer Disease Assessment Scale–Cognitive test (ADAS-Cog12) at 6 months (p = 0.014, mean =  − 3.782, 95% lower and upper confidence intervals =  − 6.79, − 0.776) which strengthened after 18 months of treatment (p = 0.018, mean − 5.782, 95% confidence interval − 10.551, − 2.388). Insulin treatment also improved CSF Aβ42/Aβ40 and Aβ42/T-tau ratios. Further, the device cohort that showed clinical benefit with insulin treatment also showed reduced progression of white matter hyperintensity volume^13^, a pathology that has been linked to inflammation and vascular injury either by amyloid or other factors^14,15^. These findings highlight the need for additional research identifying the mechanisms through which INI may provide therapeutic benefit in AD.

AD is characterized by chronic inflammation which has been postulated to be a third core marker for the disease^16^. The neuroinflammatory process described in AD is characterized by strong activation of the innate immune system, in which microglia play a central role as the primary resident macrophages in the brain^17^. CSF and serum concentrations of immune markers differ between AD and cognitively normal age matched controls^18^. Immune alterations are observed before clinical symptoms in AD mouse models, suggesting that targeting this group of cells could benefit adults with AD and perhaps attenuate cognitive decline^19^.

Another hypothesized contributing factor to AD is vascular damage and dysfunction. Much is still unknown about the complex interplay between Aβ, tau, inflammation, and vascular dysfunction^20^, but as the disease progresses the brain falls into a state of regional chronic hypoperfusion^21^. Damage to the brain’s vascular function correlates with Aβ-mediated cytotoxicity, impaired BBB functioning, reduced Aβ clearance, and an increase in inflammation^22^. Insulin has the potential to improve vascular function through a number of mechanisms including effects on vasoreactivity, inflammation, and lipid metabolism^3^.

AD pathology and related vascular dysfunction are thought to contribute to increased white matter hyperintensity volume (WMHV) as AD progresses, although the best methods of measurement are still being debated^23,24^. WMHV reflects white matter integrity which can become compromised through several pathways. It is still unknown whether cerebrovascular pathology reflected by WMHV precedes Aβ and tau, or is a consequence of their aggregation^14,25,26^. However, insulin can positively impact oligodendrocytes, reduce inflammation, and improve vascular dilation, reducing the progression of WMHV and thereby preserving brain health^27^. Vascular injury-related hypoperfusion in later stages of AD contributes to widespread neuronal loss and neurodegeneration that results in reduced gray matter volume compared to age matched controls^28^. This measurement is a reliable marker for AD, and has been incorporated in the A/T/N model for AD characterization along with amyloid and tau^29^.

In the present study we examined the cohort of participants from the large phase 2 study that used the device associated with attenuated cognitive decline, better CSF AD biomarker profiles, and reduced WMHV^12,13^. In particular, we assessed the effects of INI on CSF markers of inflammation, immune function, and vascular function and their associations with clinical markers of AD progression: CSF Aβ and tau, magnetic resonance imaging (MRI) measures, and cognitive and functional performance. Insulin acts on each of these pathways in some capacity, poising insulin as a linchpin in the cascading events of AD.

## Results

### Participants

**Figure 1:**
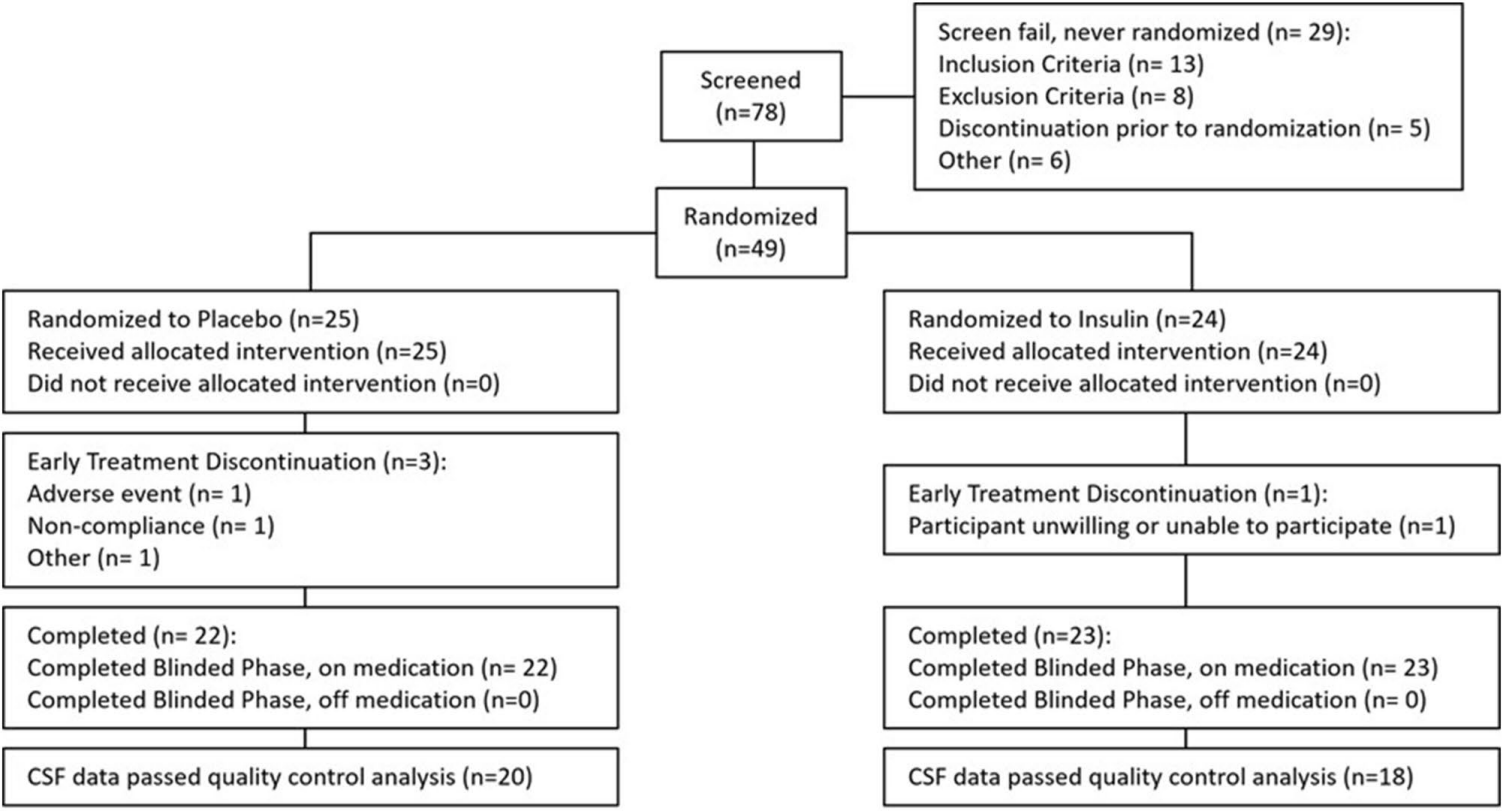
CONSORT diagram.

The parent study screened 78 participants for participation using the device that was found to be associated with clinical benefit, of whom 49 (32 men [65.3%]) were eligible and were enrolled in the study. These participants were randomized on a 1:1 ratio to either the insulin (n = 24) or placebo (n = 25) arm (Fig. 1). CSF was successfully collected at baseline and 12-month follow-up for 38 participants, whose data were analyzed for this study. There were no demographic or other notable clinical differences between those with usable and unusable data or between treatment arms at baseline (Table 1).

**Table 1:** Demographic and clinical characteristics of the study sample.

	Placebo *n* = 20	Insulin *n* = 18	Combined *n* = 38
**Gender**			
Male	13 (65%)	10 (55%)	23 (61%)
Female	7 (35%)	8 (45%)	15 (39%)
**Diagnosis**			
AD	15 (75%)	10 (55%)	25 (66%)
MCI	5 (25%)	8(45%)	13 (34%)
**APOE-ε4 carriage**			
No	5 (25%)	4 (22%)	9 (24%)
Yes	15 (75%)	14 (78%)	29 (76%)
Age, years (SD)	71.58 (7.7)	69.94 (6.12)	70.72 (6.87)
Education, years (SD)	17.2 (2.48)	16.05 (2.87)	16.62 (2.71)
Baseline ADAS-Cog	24.13 (6.46)	25.11 (9.17)	24.64 (7.91)
Baseline ADCS-ADL	43.25 (7.89)	38.89 (7.36)	40.94 (7.82)
Baseline CDR-SOB	2.69 (1.42)	3.25 (1.72)	2.98 (1.59)

### Treatment effect on CSF analytes

**Figure 2:**
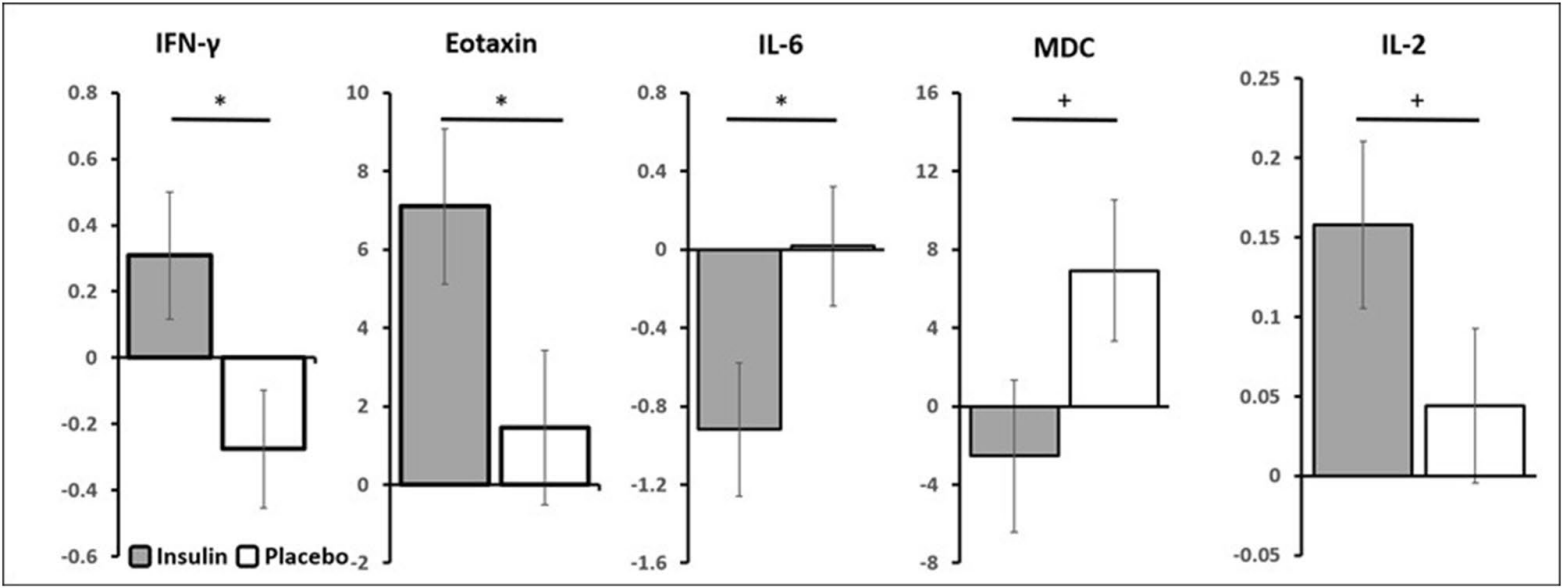
Longitudinal change in CSF analytes. Change in concentrations from baseline to month 12 differed between the insulin treated and placebo groups for CSF IFN-γ, eotaxin, and IL-6, with trends noted for MDC and IL-2. All analytes measured in pg/ml. *p < 0.05; ^+^p = 0.10 to 0.05.

CSF analytes examined for the present study are listed in Table 2. Means for all analytes are presented in Supplementary Table 1. Briefly, analyte concentrations were analyzed by repeated measures analysis of variance, with baseline concentration of the analyte, cognitive diagnosis, age, ApoE4 status, baseline MMSE, and sex as covariates as described in “Methods” section. Interactions between treatment arm and time were observed for IFN-γ, eotaxin, and IL-6 (ps = 0.032, 0.049, and 0.048). For ease of depiction, least squares mean adjusted change scores and standard deviations are presented in Fig. 2. CSF IFN-γ concentrations increased over the course of the trial in the insulin-treated group while decreasing in the placebo group. CSF eotaxin concentrations increased in the insulin-treated group to a greater degree than in the placebo group. CSF IL-6 decreased in the insulin-treated group and remained stable in the placebo group. CSF MDC and IL-2 were also found to have trending interactions (ps = 0.083 and 0.093, Fig. 2); MDC increased in the placebo group and decreased slightly in the insulin group, while IL-2 increased more in the insulin group than in the placebo group.

**Table 2:** Clinical, imaging and CSF AD variables from the parent trial, and newly analyzed CSF vascular, inflammatory and immune markers.

Cognitive and functional tests	Imaging measures	CSF Aβ, Tau and P-tau 181	Vascular markers	Inflammation and immune markers	
ADL	Deep WMHV	Aβ 40	CRP	IL-1α	Eotaxin
ADAS-Cog13	Global WMHV	Aβ 42	ICAM1	IL-1β	Eotaxin-3
CDR-SOB	Entorhinal GMV	P-tau 181	SAA	IL-2	IP-10
Memory composite	Hippocampal GMV	T-tau	VCAM-1	IL-5	MCP-1
	Temporal-parietal GMV meta-ROI	Aβ 42/40 ratio	BFGF	IL-6	MCP-4
		Aβ 42/T-tau	FLT-1	IL-7	MDC
			PIGF	IL-8	MIP-1α
			Tie-2	IL-10	MIP-1β
			VEGF-C	IL-13	TARC
			VEGF-D	IL-17A	IL12:IL23p40
			VEGF	IL-15	IFN-γ
				IL-16	TNF-α
					TNF-β

**Figure 3:**
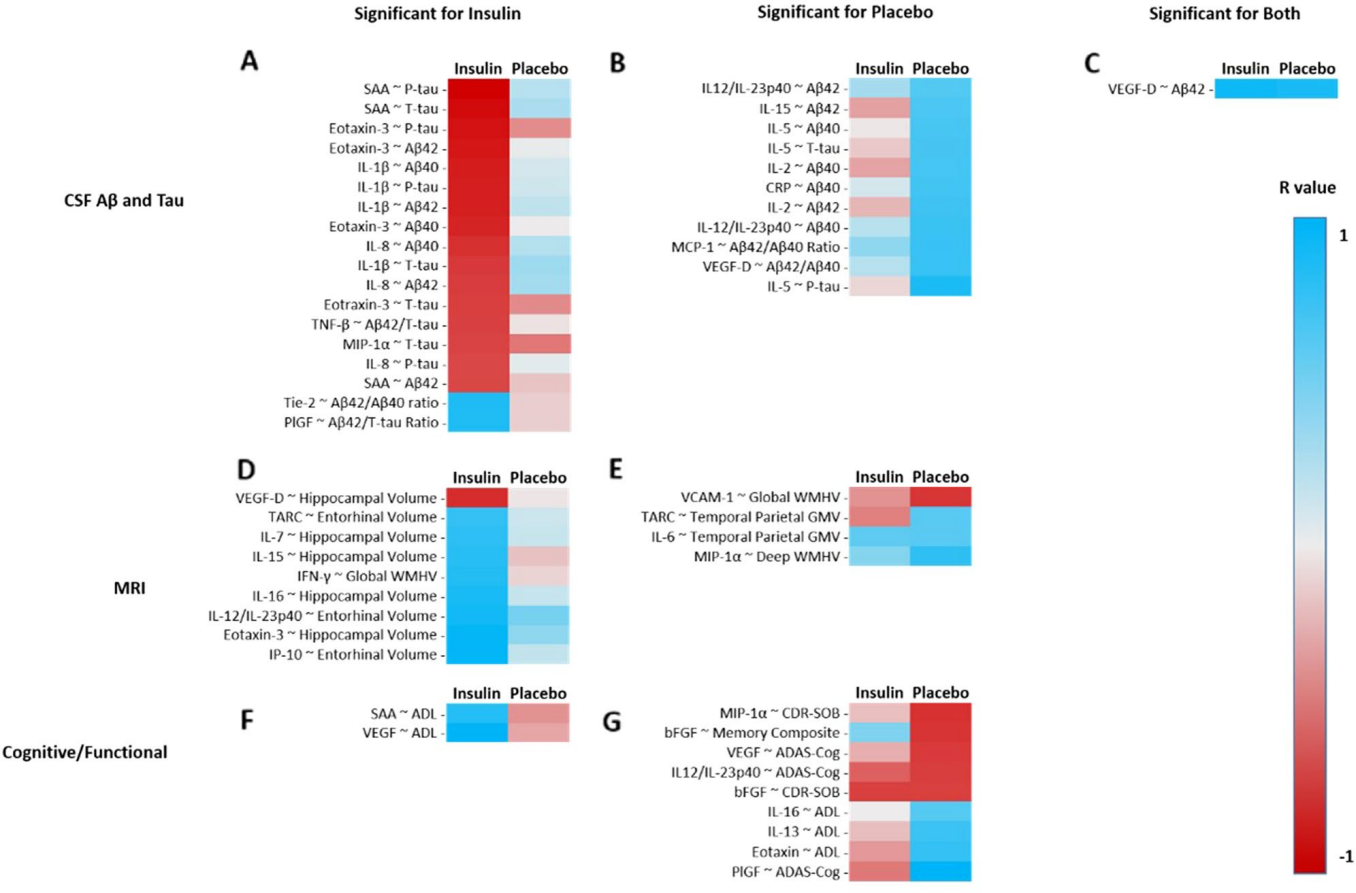
Heat map of all nominally significant (p < 0.05) associates between changes (12 month-baseline) in CSF markers of inflammation, immune function, and vascular function with changes (12 month-baseline) in markers of AD. Correlation between changes CSF markers of inflammation, immune function, and vascular function and changes in (**A,B**) CSF Aβ and tau; (**D,E**) MRI measurements; and (**F,G**) cognitive and functional tests for insulin treated and placebo groups. A single correlation only (**C**) was nominally significant for both insulin and placebo groups.

Next, we explored relationships between changes in CSF immune, inflammation, and vascular markers, with key study outcomes: (1) CSF markers of AD (Aβ40, Aβ42, Aβ42/40 ratio, total tau, tau-p181, Aβ42/tau ratio), MRI measurements of AD (global WMHV, deep WMHV, hippocampal and entorhinal cortex volume, temporal-parietal meta-region of interest), and performance on cognitive and functional tests (Alzheimer’s Disease Assessment Scale-Cognition version 13/ADAS-Cog13, memory composite, Activities of Daily Living-MCI version/ADL-MCI, Clinical Dementia Rating Scale-Sum of Boxes/CDR-SB). As detailed below, the insulin and placebo groups showed distinctly different patterns of associations between immune/inflammatory/vascular markers and study outcomes. These patterns are depicted in a heatmap in Fig. 3, which includes all observed nominally significant associations (all ps < 0.05). Due to the exploratory nature of this analysis no correction was made for multiple comparisons.

### Immune response and inflammation

#### CSF markers of AD

For the insulin-treated group (Fig. 3A), changes in eotaxin-3 were negatively correlated with changes in Aβ42, Aβ40, T-tau, and P-tau (r =  − 0.67, p = 0.004; r =  − 0.63, p = 0.008; r =  − 0.55, p = 0.027; r =  − 0.70, p = 0.002). Negative correlations were also observed between changes in MIP-1α and T-tau, as well as between changes in TNF-β and Aβ42/T-tau ratio (r =  − 0.54, p = 0.037; r =  − 0.55, p = 0.028). The insulin group also showed negative correlations between changes in IL-1β and Aβ42, Aβ40, T-tau, and P-tau (r =  − 065, p = 0.007; r =  − 0.66, p = 0.005; r =  − 0.57, p = 0.021; r =  − 0.65, p = 0.006). IL-8 changes negatively correlated with changes in Aβ42, Aβ40, and P-tau (r =  − 0.56, p = 0.023; r =  − 0.6, p = 0.015; r =  − 0.53, p = 0.035).

For the placebo group (Fig. 3B), positive correlations were observed between changes in MCP-1 and Aβ42/40 ratio, IL-12/IL-23p40 and both Aβ42 and Aβ40, IL-15 and Aβ42, and IL-5 and Aβ 40, T-tau, and P-tau (r = 0.55, p = 0.023; r = 0.48, p = 0.42; r = 0.54, p = 0.019; r = 0.5, p = 0.035, r = 0.51, p = 0.029; r = 0.52, p = 0.028; r = 0.62, p = 0.006). Positive correlations were also observed between changes in IL-2 and Aβ42 and Aβ40 (r = 0.54, p = 0.022; r = 0.52, p = 0.027).

#### MRI measurements of AD

For insulin-treated participants (Fig. 3D), hippocampal volume change positively correlated with eotaxin-3, IL-15, IL-16, and IL-17, while entorhinal volume change positively correlated with IP-10 and TARC (r = 0.65, p = 0.006; r = 0.55, p = 0.029; r = 0.59, p = 0.017; r = 0.52, p = 0.036; r = 0.65, p = 0.007; r = 0.51, p = 0.045). The insulin-treated group also showed a positive correlation between change in global WMHV and IFN-γ (r = 0.57, p = 0.038).

The placebo group (Fig. 3E) showed positive correlations between changes in deep WMHV and MIP-1alpha, as well as between temporal-parietal GMV meta-ROI and TARC (r = 0.57, p = 0.017; r = 0.47, p = 0.049; r = 0.51, p = 0.031). Positive correlations were also observed between change in temporal-parietal GMV meta-ROI and IL-8 (r = 0.47, p = 0.049).

#### Cognitive and functional tests

For the insulin group (Fig. 3F), ADAS-Cog change negatively correlated with change in MDC (r =  − 0.63, p = 0.012). In the placebo group (Fig. 3G) a negative correlation was observed between changes in ADAS-Cog and IL-12/IL-23p40, as well as between CDR-SOB and MIP-1alpha (r =  − 0.49, p = 0.041; r =  − 0.53, p = 0.024). Positive correlations were observed between change in ADL and both eotaxin and IL-16 (r = 0.56, p = 0.02; r = 0.48, p = 0.046). A positive correlation was also observed between changes in ADL score and IL-13 (r = 0.54, p = 0.025).

### Vascular injury

Next, we examined relationships between changes in CSF markers of vascular injury and changes in CSF markers of AD, MRI measurements of AD, and performance on cognitive and functional tests.

#### CSF markers of AD

In the insulin-treated group (Fig. 3A) positive correlations were observed between changes in PIGF and Aβ42/T-tau ratio, and Tie-2 and Aβ42/40 ratio (r = 0.54, p = 0.032; r = 0.57, p = 0.022). Negative correlations were observed between change in SAA and change in three CSF markers: Aβ42, T-tau, and P-tau (r =  − 0.53, p = 0.044; r =  − 0.71, p = 0.003; r =  − 0.76, p = 0.001).

In both the insulin-treated and placebo groups, changes in VEGF-D correlated with Aβ42 changes (insulin r = 0.62, p = 0.013; placebo r = 0.59, p = 0.01). This was the only nominally significant correlation demonstrated by both groups.

#### MRI measurements of AD

The insulin group (Fig. 3D) showed a negative correlation between changes in hippocampal volume and VEGF-D (r =  − 0.61, p = 0.016). In the placebo group (Fig. 3E), a negative correlation was observed between change in VCAM-1 and global WMHV (r =  − 0.51, p = 0.036).

#### Cognitive and functional tests

The insulin group (Fig. 3F) showed positive correlations between changes in ADL and SAA and between the memory composite score and VEGF (r = 0.55, p = 0.033; r = 0.67, p = 0.009). The placebo group (Fig. 3G) showed negative correlations between changes in CDR-SOB and bFGF and memory composite score, and correlations between changes in ADAS-Cog and both PlGF and VEGF (r =  − 0.48, p = 0.044; r =  − 0.52, p = 0.047; r = 0.70, p = 0.003; r =  − 0.5, p = 0.036).

## Discussion

In this retrospective study, we compared the effects of 12 months of treatment with intranasal insulin versus placebo on CSF markers of immune function, inflammation, and vascular injury in a trial cohort for whom insulin treatment was associated with beneficial effects on clinical outcomes and AD CSF and imaging biomarker profiles. Insulin treatment increased concentrations of CSF IFN-γ and eotaxin, with a similar trend for IL-2. In contrast, IL-6 was lowered with insulin treatment compared to placebo, and MDC tended to increase in the placebo group while remaining stable in the insulin group. In exploratory analyses, we then examined relationships between changes in CSF markers of inflammation, immune function and vascular integrity and clinical/AD biomarker outcomes, reasoning that correlated changes with insulin treatment might reflect underlying mechanisms related to its beneficial effects, whereas correlated changes in the placebo group might reflect the natural progression of AD. Supporting this dichotomy, we observed strikingly distinct patterns of associations in the two groups. Although our results should be considered exploratory and in need of future validation, they suggest that INI may promote a beneficial compensatory immune response that moderates the progression of AD pathology and symptoms.

We first examined whether insulin treatment changed levels of CSF immune, inflammation, and vascular markers during the 12 month treatment period compared with placebo. We found that INI increased IFN-γ, whereas IFN-γ concentrations decreased for the placebo group. Modulation of IFN-γ may have wide-ranging impact, given that it plays a critical role in orchestration of the innate and adaptive immune systems, in part through effects on microglial immune function and surveillance^30^. While one might assume that markers of increased inflammation would be associated with faster AD progression, recent reports have described the opposite relationship: higher levels of IFN-γ and other cytokines were associated with slower AD progression^31,32^. INI may thus be raising IFN-γ concentrations to levels associated with slowed AD pathology. Insulin has been shown to directly stimulate IFN-γ production by CD8 + T cells^33^; these cells are found in CSF where they provide immune protection, and have also been documented in human brain in white matter, located primarily in the perivascular space^34^. These locations provide ready access to stimulation by intranasal insulin which raises CSF insulin levels and which follows perivascular channels via bulk flow, bypassing the blood brain barrier. Our findings raise the possibility that insulin-induced increases in IFN-γ concentrations in the CNS may provide neuroprotection and attenuate cognitive decline.

We also observed that INI administration increased CSF eotaxin concentrations. Eotaxin is upregulated in the presence of inflammation and increases in both CSF and plasma with age^35^; however, its role in the brain and its relationship to AD progression are complex. Although a small study reported no differences in CSF eotaxin concentrations between normal adults and those with AD^36^, a recent finding demonstrated that, similar to IFN-γ, higher CSF eotaxin concentrations were associated with slower cognitive decline in AD^31^. In the CNS, eotaxin is produced in choroid plexus epithelial cells in response to IFN-γ, as well as by activated astrocytes surrounding blood vessels. As with IFN-γ, both loci would be readily accessible to INI; further, the effects of eotaxin are thought to be modulated by IFN-γ, such that higher levels move eotaxin in an anti-inflammatory direction^37^. In vitro, eotaxin binding to its cognate receptor CCR3 promotes angiogenesis through the AKT/PI3K pathway, one of the primary insulin signaling pathways and thus its effects may be amplified by INI^38^. In a rodent model of multiple sclerosis, eotaxin was associated with increased BBB functioning, implicating this chemokine in vessel health, as well as with increased proliferation of oligodendrocyte proliferation and remyelination^39^. These results may have relevance for human studies of ischemic stroke, in which higher eotaxin levels predicted smaller stroke volume and better functional outcomes 3 and 12 months post-stroke^40^. Higher levels of CSF eotaxin have also been associated with slower functional decline in patients with amyotrophic lateral sclerosis^41^. Thus insulin-induced increases in eotaxin may benefit multiple pathways of relevance to AD and other CNS disorders.

The cytokine IL-2 showed trend increases in response to INI treatment, and is also closely interrelated with IFNγ^42^. It is produced by CD4+ and CD8+ T cells which as noted have been documented in CSF and perivascular white matter; it is also produced by astrocytes and neurons. In APP/PSE1 mice, AAV-induced IL-2 elevations resulted in diverse beneficial effects including recruitment of astrocytes to amyloid plaques, reduced brain Aβ42/Aβ40 ratio and overall amyloid load, and improved spine density, synaptic plasticity, and memory^43^. Higher CSF IL-2 levels have been associated with slower cognitive decline in patients with MCI, leading to suggestions that IL-2 might be explored as a potential treatment for AD^44^. Clinical trials have targeted IL-2 for treatment of other diseases and confirmed its anti-inflammatory effects, which have been described as shifting the immune balance towards regulation over inflammation, and preserving T cell homeostasis^45,46^.

In addition to inducing increases in anti-inflammatory cytokines, we observed that insulin treatment reduced CSF levels of IL-6, a cytokine with multi-faceted roles in the transition from innate to acquired immunity, as well as in inducing neurotrophic responses^47,48^. It is expressed in astrocytes and endothelial cells in response to elevation in other cytokines such as IL-1β, and neuronal expression is increased in response to glutamate in rodent models of ischemia and epilepsy^48^. IL-6 has been proposed to have pro-inflammatory effects in AD; Aβ induces IL-6 expression in both astrocytes and microglia in vitro^49^, and in hippocampal neurons both Aβ and IL-6 induce synaptic dysfunction^50^. Several studies examined whether levels of CSF IL-6 are elevated in AD, but have produced conflicting results^51–54^. Given the multiple roles played by IL-6, and its complex signaling and trans-signaling activity future investigations will be needed to determine the mechanisms through which insulin may have lowered levels, and how this reduction may have impacted disease progression.

We also found that macrophage-derived chemokine (MDC, CCL22) concentrations tended to increase in the placebo group and decreased slightly with INI treatment. MDC and its receptor CCR4 play a role in inflammation and homeostasis; reduced microglia activation in mice with a TREM2 genetic mutation that increases risk of AD is accompanied by increased MDC concentrations^55^. However, MDC has not been well characterized in the context of human AD, with very few studies assessing CSF levels. Interestingly, in a previous study of participants with MCI and AD, CSF MDC concentrations were modulated by treatment with resveratrol^56^.

Taken together, we observed increases in several markers of inflammation and immune function in the insulin-treated group, who showed clinical benefit on measures of cognition and daily function and improved AD biomarker profiles in the original trial. These observations are for the most part consonant with observational studies, which have generally reported increased levels of cytokines such as IFN-γ and eotaxin to be associated with slower progression in AD. At first glance, such associations may appear counter-intuitive. However, an emerging conceptualization of inflammation and immune function as not intrinsically harmful or beneficial has emerged, with greater understanding that the impact of such changes depends on the concentrations of molecules, their location, their co-occurrence with other inflammatory/immune molecules, and with other influential aspects of the neurobiological milieu. Accordingly, a concept has emerged of a compensatory immune response system invoked by chronic inflammation in CNS disorders^57^; higher levels of such markers may reflect individual capacity to mount such a response, which in turn may enable resilience to pathological changes resulting in slower clinical progression. The anti-inflammatory effects of insulin have been noted in multiple peripheral systems, linked to inhibition of NFkβ and the NLRP3 inflammasome^58,59^. Our results suggest that intranasal insulin treatment may invoke these anti-inflammatory properties in the CNS, with corresponding therapeutic benefit in AD.

In subsequent exploratory analyses we examined the relationships between changes in markers of immune function, inflammation, and vascular integrity and changes in key outcomes from the original trial, including classical AD CSF biomarkers, imaging biomarkers, and cognitive/functional measures for the insulin-treated and placebo groups. A striking difference in the overall pattern of relationships in the two groups emerged; the insulin and placebo groups had nearly completely different associations, with only one correlation overlapping between groups. This divergence suggests that INI might alter the typical progression of AD as demonstrated by the placebo group.

Inspection of specific correlations showed frequent associations involving several markers of inflammation and immune function in the insulin-treated group. IL-1β has been found to be elevated in the CSF of adults with AD^18^. In the insulin-treated group, increased IL-1β, eotaxin-3 and IL-8 were associated with reduced Aβ42, Aβ40, and P-tau. A speculative interpretation of these relationships is that insulin treatment enhanced the immune/inflammatory response which in turn improved microglial phagocytosis and degradation of amyloid, preventing increased CSF concentrations, and amyloid-induced elevations in P-tau. This possibility is also consistent with the finding that increased eotaxin-3 levels correlated with less loss of hippocampal gray matter volume.

Several markers of vascular injury were also related to outcomes from the parent trial for the insulin-treated group. Serum amyloid A (SAA) is secreted in response to inflammation and associated with damage to blood vessels^60^. We found that decreased SAA following insulin treatment correlated with greater preservation of function measured by the ADL-MCI scale. We also found that insulin-induced increases in VEGF, a growth factor that stimulates blood vessel health, angiogenesis, and wound repair^61^, predicted better performance on a memory composite score. Two other markers of angiogenesis, tyrosine kinase-2 (Tie-2) and placental growth factor (PlGF), positively correlated with Aβ42/40 and Aβ42/T-tau ratios respectively. PlGF is notable for its involvement in maturation and stabilization of blood vessels under hypoxic conditions. Both PlGF and Tie-2 have been shown to modulate BBB function^62,63^; increased blood vessel stability could enhance clearance of Aβ42 from the brain and into the CSF. Although not well-studied in AD, one study has reported increased concentrations in CSF PlGF from adults with AD or other neurodegenerative disorders, and a second showed lower levels of Tie2 in AD^64,65^. As the BBB is known to be disrupted in AD, insulin-induced modulation of PlGF and Tie-2 could improve BBB function and thereby aid in the clearance of Aβ.

Our study has several limitations. It is an exploratory study with post hoc analyses in a small sample and therefore not powered to account for multiple comparisons. Future larger studies are needed to validate changes in these neuroinflammatory/immune/vascular biomarkers in response to intranasal insulin treatment and examine their relationship to AD progression. Larger studies are also needed to further understand inter-relationships among these markers, perhaps through application of more sophisticated multivariate or machine learning approaches. Although our results demonstrate robust effects in response to 12 months of INI administration, an important question remains concerning the brain’s response to longer insulin exposure. Despite these weaknesses, we believe these findings provide strong rationale for future examination of INI in AD and MCI using well-validated delivery technologies.

In conclusion, our results showed that intranasal insulin treatment modulated markers of immune function, inflammation, and vascular integrity in the cohort who showed clinical benefit in the parent trial in a manner that suggests activation of a compensatory immune response system. Within the insulin-treated group, a pattern of associations was observed between changes in these markers and changes in CSF, imaging and clinical outcomes from the parent trial that diverged strikingly from patterns observed for the placebo group, suggesting that insulin is altering the progression of AD in fundamental ways. Our results also highlight the complexity of interpretation of immune/inflammatory profiles, in which negative or beneficial effects are influenced by the neurobiological milieu at each stage of disease. Further research is needed to identify the pathways through which these immune/inflammatory/vascular mediators act and the specific mechanisms through which they are affected by insulin. Finally, our findings provide additional evidence that INI is a promising therapeutic option for AD.

## Methods

This study consisted of a 12-month placebo-controlled double-blinded phase, in which participants were randomized on a 1:1 basis to receive 40 IU of intranasal insulin (20 IU b.i.d. Humulin-R U100) or placebo (insulin diluent) followed by a 6-month open label extension of 40 IU insulin. The trial was overseen by the Alzheimer’s Therapeutic Research Institute (P. Aisen, Director) together with the Principal Investigator (S. Craft). The trial was registered with ClinicalTrials.gov (NCT01767909; first date of registration 15/01/2013). Eligibility and recruitment methods for this study have been described elsewhere^12^. The parent study used two devices to deliver insulin intranasally. The initial device for the trial had been used in previous INI studies, but new adaptations introduced by the manufacturer caused sporadic reliability issues, resulting in compliance that was slightly less than the desired 80% target (compliance for both placebo and insulin groups averaged about 73%); thus a second device from a different manufacturer was adopted after trial onset which had not been used in previous AD studies. However, only the cohort using the initial device demonstrated changes in AD CSF biomarkers and cognitive benefits over the 18-month long study, and the cohort that used that device is the subject of these retrospective analyses. Study protocols and informed consent were approved by the institutional review boards at the individual study sites which were all academic research centers, and at the University of California, San Diego, the University of Southern California, and the Wake Forest School of Medicine. All methods were performed in accordance with relevant guidelines and regulations. Written informed consent was obtained from participants and study partners. The study was conducted under local institutional review board supervision. Briefly, participants with amnestic MCI (n = 18) or AD (n = 31) were recruited from 12 sites. Participants received baseline testing including the ADAS-Cog13, Mini-Mental State Exam (MMSE), Clinical Dementia Rating (CDR), a lumbar puncture, and an MRI, then were randomized on a 1:1 basis to receive either placebo (n = 25) or 20 IU intranasal insulin (n = 24) twice daily for 12 months using a weighted algorithm as previously described^12^. After 12 months the cognitive battery was readministered, another lumbar puncture was performed, and another MRI was obtained. A total of 38 participants (placebo n = 20; insulin n = 18) with CSF data that passed quality control measures at baseline and month 12 were included in analyses.

### Imaging biomarkers

T1 and Fluid Attenuated Inversion Recovery images were collected with 1.5 or 3 T MRI. FreeSurfer 6.0.0 was used to produce participant-specific gray matter volume by processing T1 weighted images. FLAIR images were segmented by the lesion growth algorithm^66^ as implemented in the LST toolbox version 3.0.0 (www.statisticalmodelling.de/lst.html) for SPM. The algorithm first segments the T1 images into the three main tissue classes (CSF, GM and WM). This information is then combined with the coregistered FLAIR intensities in order to calculate lesion belief maps. By thresholding these maps with a pre-chosen initial threshold (κ = 0.3) an initial binary lesion map is obtained which is subsequently grown along voxels that appear hyperintense in the FLAIR image. The result is a lesion probability map. The lesion probability maps were then warped to MNI space and lobar volume was extracted using Mayo Clinic Adult Lifespan Template^67^. Global WMHV was then calculated by excluding brainstem and cerebellum.

T1 weighted images were used to analyze gray matter volume. Bilateral entorhinal, inferior temporal, middle temporal, inferior parietal, fusiform, and precuneus were used to construct a temporal-parietal GMV meta-ROI^68^. Hippocampal and entorhinal volumes were extracted and analyzed individually.

### CSF biomarkers

CSF was collected in the morning after an overnight fast. CSF aliquots were immediately placed on dry ice and either stored at − 80 °C for later analysis or shipped overnight to a central biomarker laboratory for analysis of AD biomarkers Aβ42, Aβ40, total tau, and tau phosphorylated at threonine 181 using the Meso Scale Discovery platform as previously described^12^. Neuroinflammation/immune/vascular markers were assessed *en bloc* on first thawed CSF samples using Meso Scale Discovery Neuroinflammation Panel 1 multiplex kits on a MESO Quickplex SQ 120 (Meso Scale Diagnostics, Rockville, MD, USA) at the Wake Forest School of Medicine. This kit is a compilation of 5 individual multiplex kits as follows: Cytokine Panel 1, Chemokine Panel 1, Proinflammatory Panel 1, Vascular Injury Panel 2, and Angiogenesis Panel 1. These panels included 37 analytes which have been identified as frequently abnormal using human CSF from AD, Parkinson’s Disease and other neurological disorders, as well as healthy controls, and is promoted as a tool for discovery (for a description of this panel and its development, see https://www.mesoscale.com/~/media/files/product%20inserts/neuroinflammation%20panel%201%20human%20insert.pdf). Protocols were modified to extend the standard calibration curve to increase the number of samples with analyte concentrations above the lower limit of detection. Reliably detectable concentrations could not be obtained for IL-4 which was then eliminated from further analysis. Remaining analytes are listed in Table 2 and were grouped into 2 categories: inflammatory/immune markers (Eotaxin [CCL11], Eotaxin-3 [CCL26], interferon gamma induced protein 10 [IP-10, CXCL10], monocyte chemoattractant protein-1 [MCP-1 , CCL2], monocyte chemoattractant protein-4 [MCP-4, CCL13], macrophage-derived chemokine [MDC, CCL22], macrophage inflammatory protein-1α [MIP-1α, CCL3], MIP-1β [CCL4], thymus and activation-regulated chemokine [TARC, CCL17], IL-12:IL-23p40, IL-15, IL-16, IL-17A, IL-1α, IL-5, IL-7, and tumor necrosis factor beta [TNF-β]) and inflammation (Interferon-gamma [IFN-γ], IL-10, IL-13, IL-1β, IL-2, IL-6, IL-8, tumor necrosis factor alpha [TNF-α]), and vascular markers (C-reactive protein [CRP], intercellular adhesion molecule-1 [ICAM-1], serum amyloid A [SAA], vascular cell adhesion molecule-1 [VCAM-1], basic fibroblast growth factor [BFGF], fms-like tyrosine kinase 1 [FLT-1], placental growth factor [PIGF], tyrosine kinase-2 [Tie-2], vascular endothelial growth factor [VEGF], VEGF-C¸ VEGF-D). Mean intra-assay CVs for internal control analytes were as follows: Cytokine Panel 1 (4.2%), Chemokine Panel 1 (4.7%), Proinflammatory Panel 1 (9.3%), Vascular Injury Panel 2 (2.4%), and Angiogenesis Panel 1 (3.7%). Analyte concentrations below the LLOD were assigned a value of one half the value of LLOD. APOE genotyping was performed on DNA isolated from whole blood using an established protocol and participants were dichotomized into E4 carriers and non-carriers^12^.

Cognitive and functional testing consisted of the ADAS-Cog, CDR-SOB, Alzheimer Disease Cooperative Study ADL, and a memory composite defined as the sum of z scores from the Free and Cued Selective Reminding Test and immediate and delayed story recall^69–71^. Together cognitive and functional tests, CSF Aβ and Tau concentrations, and MRI measurements of WMHV and GMV are referred to as markers of AD progression.

Cross sectional analysis was performed to assess group differences at baseline in age, cognitive status, and sex using chi squared tests or general linear models when appropriate. Change in GMV and WMHV were constructed as previously described^13^. In the first phase of analysis, to determine whether there were treatment-related changes in neuroinflammatory/immune/vascular markers over the 12 month treatment period, general linear modeling was performed in SAS v 9.4 to conduct repeated measures analysis of variance, with treatment group (insulin vs. placebo) as the independent factor and time (baseline vs. 12 months) as the within group factor, and with inclusion of the following covariates: baseline concentration of the analyte, cognitive diagnosis, age, ApoE4 status, baseline MMSE, and sex. Non-contributing covariates (p > 0.15) were dropped from the model. Following the identification of a significant time by treatment interaction, post hoc comparisons of least square adjusted means were conducted. In the second exploratory phase of analysis, Pearson’s r correlations were used to determine relationships between changes in neuroinflammatory/immune/vascular variables, and cognitive and AD biomarker changes separately for insulin and placebo groups. Due to the exploratory nature of the analyses, no corrections for multiple comparisons were applied, and all correlation p values < 0.05 were considered nominally significant. All correlations with p < 0.05 were then plotted in a heat map as shown in Fig. 3 to enable the appreciation of differences in correlation patterns between the insulin and placebo groups.

## Supplementary Information

Supplementary Table 1.
