# Supplementary material for: Intranasal insulin modulates cerebrospinal fluid markers of neuroinflammation in mild cognitive impairment and Alzheimer’s disease: a randomized trial
Source: Sci Rep. 2022 Jan 25;12:1346. doi: 10.1038/s41598-022-05165-3 (PMC8789895; doi:10.1038/s41598-022-05165-3)
Supplement: Supplementary file 1 — Supplementary Table 1. [file 41598_2022_5165_MOESM1_ESM.docx]

Supplementary Table 1. Raw means and standard deviations for immune, inflammation and vascular analytes.
